# Effects of Dietary Ferroporphyrin Supplementation on Growth Performance, Antioxidant Capacity, Immune Response, and Oxygen-Carrying Capacity in Gibel Carp (*Carassius auratus gibelio*)

**DOI:** 10.3390/ani14213104

**Published:** 2024-10-28

**Authors:** Kai Wang, Lu Zhang, Hualiang Liang, Mingchun Ren, Haifeng Mi, Dongyu Huang, Jiaze Gu

**Affiliations:** 1 College of Fisheries and Life of Science, Shanghai Ocean University, Shanghai 201306, China; 2 Tongwei Agricultural Development Co., Ltd., Key Laboratory of Nutrition and Healthy Culture of Aquatic Livestock and Poultry, Ministry of Agriculture and Rural Affairs, Healthy Aquaculture Key Laboratory of Sichuan Province, Chengdu 610093, China; 3 Key Laboratory of Integrated Rice-Fish Farming Ecology, Ministry of Agriculture and Rural Affairs, Freshwater Fisheries Research Center, Chinese Academy of Fishery Sciences, Wuxi 214081, China; 4 Wuxi Fisheries College, Nanjing Agricultural University, Wuxi 214081, China

**Keywords:** gibel carp, ferroporphyrin, antioxidant capacity, immune capacity

## Abstract

An eight-week experiment was conducted to study the effects of dietary ferroporphyrin (FPR) supplementation on growth performance, antioxidant capacity, immune response, and oxygen-carrying capacity in gibel carp. The results demonstrated that the addition of FPR increased the moisture content of the whole fish body. Supplementation with 0.01% FPR significantly increased the plasma albumin (ALB), total protein (TP), and total cholesterol (TC) contents. The addition of 0.03% and 0.04% FPR significantly increased alanine aminotransferase (ALT) and aspartate aminotransferase (AST) levels, respectively, while the glucose (GLU), TC, and total triglyceride (TG) levels showed opposite trends. In terms of antioxidant capacity, the 0.03% and 0.04% dietary FPR supplementation increased malondialdehyde (MDA) levels. The activity of glutathione peroxidase (GPx) exhibited an opposite trend to MDA levels. The supplementation of 0.03% of FPR resulted in a notable reduction in mRNA expression levels of *nrf2*, *keap1*, *cat*, and *gpx.* Regarding immunity, 0.01% FPR supplementation down-regulated the expression levels of *il-1β* mRNA, while 0.02% FPR down-regulated *il-6* and *nf-κb* expression levels. Furthermore, 0.02% FPR supplementation significantly up-regulated the *il-10* mRNA expression levels. In terms of oxygen-carrying capacity, high levels of FPR (0.03% and 0.04%) were found to influence the *epo* and *vegf* mRNA expression. In conclusion, the incorporation of dietary 0.01–0.02% FPR improved the immune system of gibel carp without affecting their antioxidant and oxygen-carrying capacity. However, supplementation with higher levels of FPR (0.03–0.04%) led to decreased antioxidant and oxygen-carrying capacity.

## Simple Summary

This study was conducted to investigate the effects of ferroporphyrin (FPR) supplementation on growth performance, antioxidant capacity, immune response, and oxygen-carrying capacity in gibel carp. Based on 8 weeks of a feeding experiment, the results of this experiment demonstrated that dietary FPR supplementation enhanced the immune response of gibel carp. Lower concentrations of dietary FPR (0.01–0.02%) down-regulated the NF-κB signaling pathway, thereby improving the immune ability without affecting their antioxidant and oxygen-carrying capacity. However, supplementation with higher levels of FPR (0.03–0.04%) inhibited the Nrf2 and HIF-1α signaling pathways, leading to decreased antioxidant and oxygen-carrying capacity.

## 1. Introduction

Fish and fishery products represent a significant source of protein for the global population. The global supply of fish food has grown significantly over the last few decades, and aquaculture is recognized as a major source of fish product supply, with global aquaculture production growing at an average annual rate of 6.7% over the period 1990–2020 [1,2]. As the standard of living has improved and requirements for aquatic products have increased, the scale and density of aquaculture have gradually increased [3]. Although high-density aquaculture enhances productivity, this method renders fish susceptible to a range of detrimental metabolic and physiological conditions, including immunosuppression and oxidative stress damage, which increases the risk of infectious diseases [4,5]. Furthermore, high-density aquaculture facilitates the rapid dissemination of pathogens, resulting in elevated mortality rates [1]. This is an industry-wide issue that needs to be addressed urgently. In the past, chemicals and antibiotics were widely employed as preventive and control measures for disease outbreaks in some countries. However, prolonged or inappropriate use of chemical and antibiotic agents may result in water contamination and the accumulation of residual pharmaceutical compounds, potentially posing a significant risk to human health [6]. With increased environmental awareness and advancements in research and technology, societal perceptions of disease prevention and control have shifted. In addition to the direct elimination of exogenous pathogens through the use of antibiotics and chemicals, it is also possible to modulate an organism’s immune system to increase its resistance to pathogen invasion [7]. Consequently, there is a mounting interest in the application of natural additives with the objective of substituting the use of antibiotics and chemicals, such as purple coneflower [8], astaxanthin [9], taurine [10,11], and glutamine [12]. A substantial body of evidence indicates that the appropriate addition of specific additives into animal feed may improve the health status of the respective animals, thereby improving their resistance to pathogen invasion and disease [13,14,15].

Ferroporphyrin (FPR), a porphyrin ring compound containing iron, is an activator of heme oxygenase (HO) enzymes and has been used to safely treat porphyria since the 1970s [16]. In addition to its role in the treatment of acute porphyrias, FPR has been found to play an essential protective role in many pathological conditions, including autoimmune disorders, tumor formation, and cardiovascular diseases by inducing Heme Oxygenase-1 (HO-1) [17]. HO-1 is a rate-limiting enzyme in the stress response pathway of FPR catabolism. HO-1 catalyzes the degradation of FPR into one of three byproducts, namely, carbon monoxide (CO), which possesses anti-inflammatory properties; biliverdin, which exhibits antioxidant functions; and iron [18,19]. Furthermore, numerous studies have indicated that FPR plays a pivotal role in a multitude of vital biological processes. For instance, FPR can be employed as an immunomodulator to attenuate the inflammatory response elicited by influenza A infection [20]. FPR has been demonstrated to reduce tissue inflammation and antioxidant damage in obese mice, as well as to treat UV-radiation-induced skin damage and apoptosis in mice [16,21]. Additionally, FPR can be employed as an additive for treatment of anemia caused by iron deficiency in both humans and animals [22]. Furthermore, FPR belongs to the porphyrin family, and, in animals, FPR is the oxidized form of heme that contains a single iron atom in the center. Heme is a ferroprotoporphyrin IX complex and exists in a range of cells, including erythroid and non-erythroid cells, and it has a variety of biological functions, including roles in oxygen transport and storage, cellular and xenobiotic metabolism, and the regulation of inflammatory factors [21,23]. Recent studies have indicated that FPR may reduce the formation of reactive oxygen species (ROS) by degrading HO-1 [24] and indirectly influence the hypoxia signaling pathway by regulating heat shock proteins [25]. Therefore, FPR may serve as a beneficial dietary additive to improve the antioxidant capacity, immune function, and oxygen-carrying capacity of fish.

The gibel carp (*Carassius auratus gibelio*), a benthic freshwater fish that belongs to the Cyprinidae family, exhibits one of the highest resistances to hypoxia compared to other fish species [26]. Due to its palatable meat, strong adaptability, and rich nutritional value, the gibel carp is the primary freshwater aquaculture species cultivated in China [27,28,29,30,31]. In 2022, the annual production of gibel carp in China was approximately 2.783 million tons [32]. As the scale and density of aquaculture expands, gibel carp are increasingly susceptible to diseases caused by pathogens, resulting in substantial economic loss to the industry [33]. An increasing number of studies have demonstrated that the incorporation of immune-system-enhancing additives into fish diets can mitigate the incidence of disease, thereby reducing losses within economics of this industry [34,35]. Furthermore, the practice of high-density aquaculture has the potential to impact various aspects of fish biology, including food competition and consumption, growth, and the generation of non-adaptive stressors that can affect the overall quality of life and welfare of the fish [36]. Consequently, to improve growth and health status, studies on the anti-stress function of feed are urgently needed. Nevertheless, the impact of FPR on gibel carp remains uncertain, which may restrict the application of FPR in aquaculture. Accordingly, the objective of this experiment was to explore the impact of FPR on the biological processes of gibel carp, including growth performance, antioxidant capacity, immune response, and oxygen-carrying capacity.

## 2. Materials and Methods

### 2.1. Preparation of Diets

Five experimental diets were prepared, and 0, 0.01%, 0.02%, 0.03%, and 0.04% FPR were added, respectively. The component combinations of each basal diet are outlined in Table 1. The individual ingredients were ground and then filtered through an 80-micron mesh. The ingredients were weighed in accordance with the dietary formulation and gradually combined with water and soy oil. Subsequently, the mixture was transferred to a pelletizer (F-26 (II), South China University of Technology, China) to produce 1 mm pellets. The prepared feed was dried at 45 °C and refrigerated until further use.

### 2.2. Experimental Fish Feeding and Managing Process

Juvenile gibel carp were provided by the Freshwater Fisheries Research Center (FFRC), Wuxi, China. The fish were fed commercial feed and adapted to the experimental environment two weeks prior to experimentation. Three hundred healthy fish with high vitality and no epidermal damage were selected. The fish had an average body weight of 36 ± 0.03 g and were randomly allocated into 15 cages (1 m × 1 m × 1 m). Three cages were assigned to each experimental group, and each cage contained 20 fish. The feeding times were at 7:00 and 17:00 daily until the fish were satiated. The weight and mortality status of the fish were monitored daily. During the feeding trial, the water temperature was maintained at 28 °C to 30 °C (thermometers), the dissolved oxygen concentration was maintained at ≥6.4 mg/L (Pen Dissolved Oxygen Meter, Dongguan Wanchuang Electronic Products Co., Guangzhou, China), and the pH exhibited fluctuations between 7.3 and 8.0 (Dr Water kit, Heachen Energy Technology Co., Ltd., Shanghai, China). The feeding experiment lasted for eight weeks. No disease outbreak occurred during the experiment.

### 2.3. Collection of Samples

After the eight-week feeding experiment, the fish were deprived of food for 24 h and then weighed. Six fish were randomly selected from each cage. Three were stored at −20 °C for fish body composition analysis, and the remaining three were utilized for the collection of liver and blood samples. Prior to sample collection, the fish were anesthetized using MS-222 (100 mg/L). Blood samples were collected from the caudal vein and centrifuged at 5000 rpm and 4 °C for 10 min (Eppendorf AG 22331 Hamburg, Germany). The plasma was collected and stored at −80 °C until further blood biochemical analysis. The abdominal cavities of the fish were cut open, and the livers were collected and stored at −80 °C until further analysis.

### 2.4. Chemical Analysis

The proximate composition (crude protein, crude lipid, crude ash, and moisture) of the diets and fish were measured by methods of the AOAC [37]. Plasma biochemical parameters including total protein (TP, Biuret method), glucose (GLU, Hexokinase method), total triglycerides (TG, Oxidase method), albumin (ALB, Bromocresol green method), total cholesterol (TC, Oxidase method), alanine aminotransferase (ALT, IFCC method), and aspartate aminotransferase (AST, IFCC method) were measured using a fully automated biochemical analyzer (Mindray BS-400, Shenzhen, China) with the corresponding Mindray kits, in accordance with the manufacturer’s protocols. The activity of liver-relevant antioxidant enzymes, including catalase (CAT, Ammonium molybdate method, #A007-1-1), superoxide dismutase (SOD, WST-1 method, #A001-3), total antioxidant capacity (T-AOC, ABTS method, #A015-2-1), and glutathione peroxidase (GPx, Microplate method, #A005-1), as well as the content of the lipid peroxidation product, such as malondialdehyde (MDA, TBA method, #A003-1), were analyzed using commercial kits (Nanjing Jiancheng Institutes, Nanjing, China) following the manufacturer’s protocols.

### 2.5. Gene Expression Analysis

Real-time PCR was used to detect the relative expression of liver genes as described in our previous study [38]. In brief, the total RNA was extracted from the liver tissue using the RNAiso Plus kit (Vazyme, Nanjing, China). The quality and concentration of the RNA (the A_260/280_ values between 1.8 and 2.0) were assessed using a spectrophotometric method on a NanoDrop 2000 spectrophotometer (Thermo Fisher Multiskan GO, Shanghai, China). Primers were designed online using Primer Premier 6.0 based on partial cDNA sequences of the genes. The specific primer sequences designed for this experiment are detailed in Table 2. Subsequently, the mRNA levels were quantified by real-time PCR on a 7500 real-time fluorescence quantitative PCR instrument (Applied Biosystems, Foster City, CA, USA) using a one-step qRT-PCR SYBR PrimeScript TM PLUS RT–PCR kit (Takara, Dalian, China). The reaction procedure was as follows: 50 °C for 15 min, 95 °C for 30 s, and then 95 °C for 10 s, followed by 60 °C for 30 s, for a total of 40 cycles. The standard curve method [39] was employed for the calculation of mRNA levels, and the reference gene selected for this study was *β-actin*. The R^2^ and PCR efficiency of the genes involved in this experiment are presented in Table 2.

### 2.6. Statistical Analysis of Data

The statistical analyses were conducted using IBM SPSS (Statistical Package for Social Sciences 26.0) software, with a one-way ANOVA and Tukey’s multiple comparison tests employed for all experimental data (means ± SE) [37]. The differences between groups were significant when *p* < 0.05. The use of different letters indicates the presence of significant differences between groups.

## 3. Results

### 3.1. Gibel Carp’s Growth Performance and Whole-Body Composition

The growth performance of each group is presented in Table 3. Gibel carp weight gain (WGR), final body weight (FW), and specific growth rate (SGR) exhibited an increased trend and then decreased in response to rising FPR levels, whereas the opposite trend was observed for the feed conversion ratio (FCR). However, no significant differences were observed between the control and any FPR diet in terms of WGR, SGR, FCR, and FW (*p* > 0.05).

The data presented in Table 4 show the whole-body composition of the gibel carp. As the proportion of dietary FPR increased, the moisture content initially rose and then exhibited a declining trend. The highest moisture content was observed at 0.03% FPR (*p* < 0.05). Supplementation with 0.03% FPR significantly increased the lipid content of the whole fish body compared to the 0.02% group (*p* < 0.05). Moreover, the addition of FPR did not affect the protein and ash content (*p* > 0.05).

### 3.2. Plasma Biochemical Indices

The results of plasma biochemical parameters are shown in Table 5. The ALT and AST contents increased with the increasing FPR levels, and the highest contents were found in 0.03% and 0.04%, respectively (*p* < 0.05). TG and GLU contents showed an opposite trend, and supplementation with 0.03% and 0.04% FPR significantly decreased TG and GLU levels (*p* < 0.05). ALB levels fluctuated, and supplementation with 0.01% FPR significantly increased the ALB content (*p* < 0.05). Compared to the control, the group fed with 0.01% FPR demonstrated the highest TC levels, whereas supplementation of 0.04% FPR showed a significant reduction of TC levels compared to the control (*p* < 0.05). As the FPR level increased, the TP levels demonstrated a tendency to ascend and subsequently decline. The highest level was observed in the 0.01% group (*p* < 0.05).

### 3.3. Antioxidant-Related Indices of Liver

**Figure 1:**
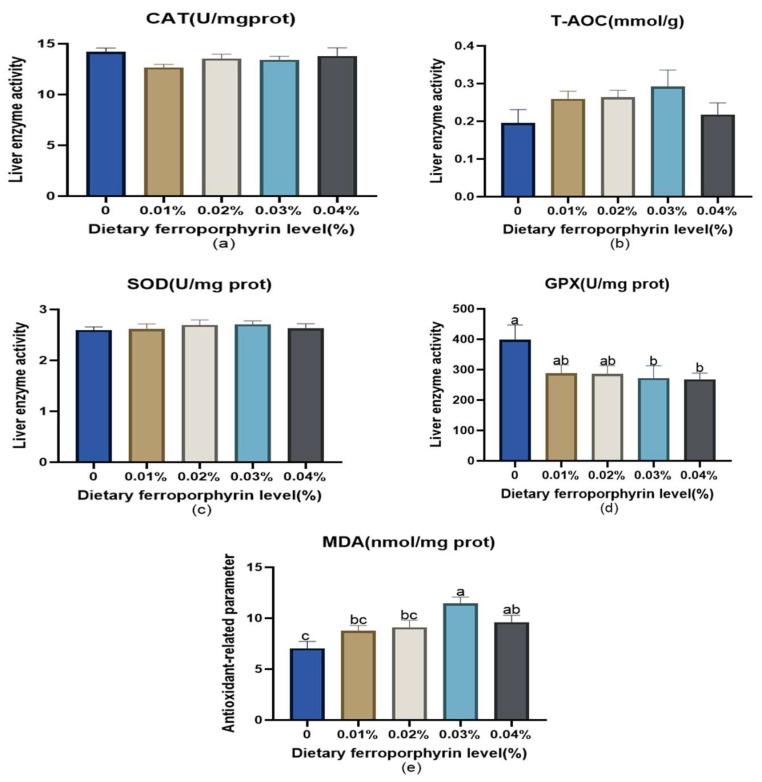
Liver antioxidant-related parameters: (**a**) catalase (CAT); (**b**) total antioxidant capacity (T-AOC); (**c**) superoxide dismutase (SOD); (**d**) glutathione peroxidase (GPx); (**e**) malondialdehyde (MDA). The appearance of superscripts in the bar with different letters denotes statistically significant disparities (*p* < 0.05), whereas the absence of superscripts signifies that the difference was not significant (*p* > 0.05).

Figure 1 presents the antioxidant-related indices of liver. Dietary supplementation of 0.03% and 0.04% FPR led to a notable elevation in MDA levels (*p* < 0.05). Conversely, supplementation with 0.03% and 0.04% FPR significantly reduced GPx levels (*p* < 0.05). It was observed that FPR supplementation at any concentration did not significantly affect the activities of T-AOC, CAT, and SOD (*p >* 0.05).

### 3.4. Expression Level of Antioxidant-Related Genes

**Figure 2:**
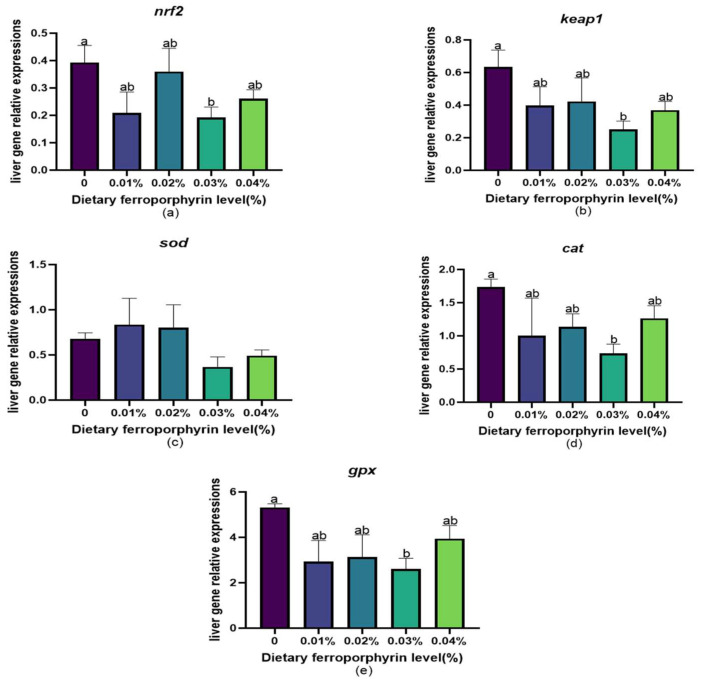
Expression level of antioxidant-related genes in the liver of gibel carp: (**a**) *nrf2*; (**b**) *keap1*; (**c**) *sod*; (**d**) *cat*; (**e**) *gpx*. The appearance of superscripts in the bar with different letters denotes statistically significant disparities (*p* < 0.05), whereas the absence of superscripts signifies that the difference was not significant (*p* > 0.05).

The results of the mRNA levels of antioxidant-related genes in the liver are shown in Figure 2. The expression levels of *keap1*, *nrf2*, *gpx*, and *cat* exhibited a significant reduction in response to the addition of FPR. The 0.03% group demonstrated the lowest expression levels of *keap1*, *nrf2*, *gpx*, and *cat* (*p* < 0.05). In addition, dietary FPR did not significantly influence the mRNA expression of *sod* (*p* > 0.05).

### 3.5. Expression Levels of Genes Involved in the NF-kB Signaling Pathway

**Figure 3:**
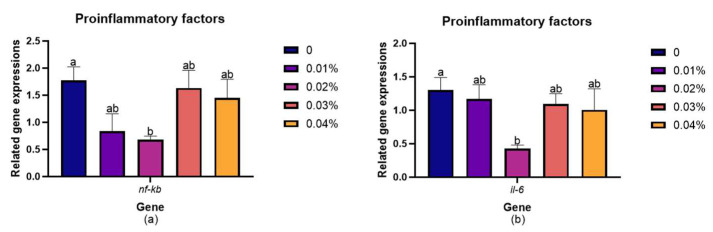

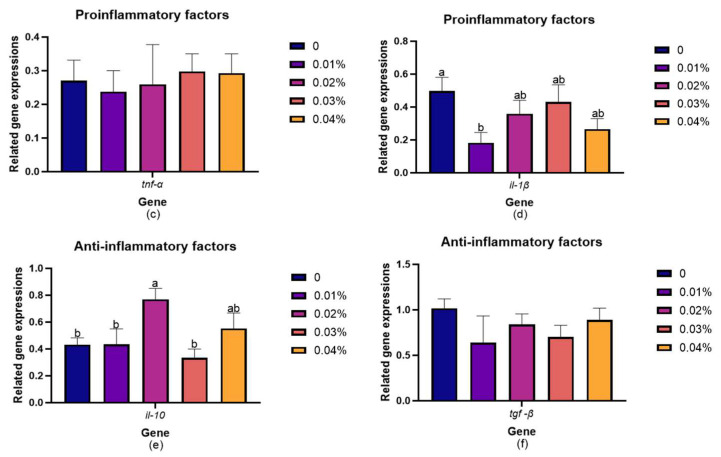
Expression level of NF-κB-signaling-pathway-related genes in the liver of gibel carp: (**a**) *nf-κb*; (**b**) *il-6*; (**c**) *tnf-α*; (**d**) *il-1β*; (**e**) *il-10*; (**f**) *tgf-β*. The appearance of superscripts in the bar with different letters denotes statistically significant disparities (*p* < 0.05), whereas the absence of superscripts signifies that the difference was not significant (*p* > 0.05).

Figure 3 represents the expression of NF-κB signaling pathway-related genes. Supplementation with 0.02% FPR significantly inhibited the expression of both *il-6* and *nf-κb* mRNA (*p* < 0.05), while supplementation with 0.01% FPR significantly reduced the expression levels of *il-1β* mRNA (*p* < 0.05). Conversely, supplementation with 0.02% FPR was observed to markedly elevate the *il-10* mRNA expression (*p* < 0.05). Furthermore, there was no notable impact on *tnf-α* and *tgf-β* mRNA expression levels as a result of elevated dietary FPR (*p* > 0.05).

### 3.6. Expression Levels of Genes Related to Oxygen-Carrying Capacity

**Figure 4:**
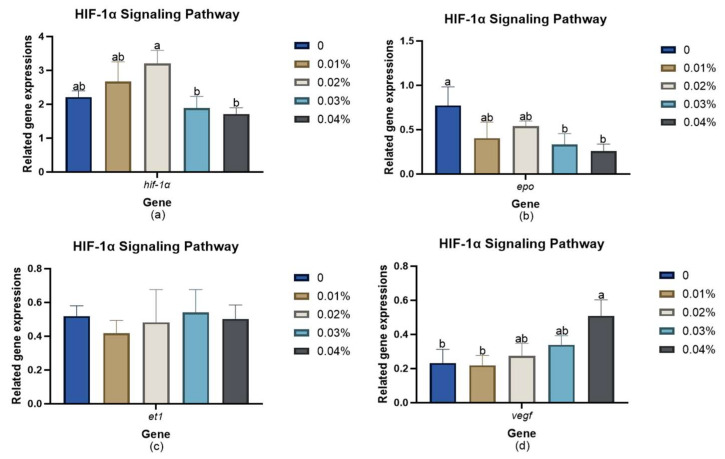
Expression level of HIF-1α-signaling-pathway-related genes in the liver of gibel carp: (**a**) *hif-1α*; (**b**) *epo*; (**c**) *et1*; (**d**) *vegf.* The appearance of superscripts in the bar with different letters denotes statistically significant disparities (*p* < 0.05), whereas the absence of superscripts signifies that the difference was not significant (*p* > 0.05).

Figure 4 shows the relative expression level of genes related to the HIF-1α signaling pathway. Initially, *hif-1α* mRNA expression demonstrated an increase in levels and then decreased as FPR concentration rose. However, *hif-1α* mRNA remained statistically indistinguishable from those observed in the control group (*p* > 0.05). The *epo* mRNA expression level exhibited a gradually decreasing trend with the lowest expression levels found in the 0.03% and 0.04% groups (*p <* 0.05). Compared to the control group, supplementation with 0.04% FPR significantly increased *vegf* mRNA expression levels (*p <* 0.05). In addition, the *et1* mRNA expression level was not affected by any dietary FPR supplementation levels (*p >* 0.05).

## 4. Discussion

### 4.1. Effects of Dietary Supplementation with FPR on the Growth Performance and Whole-Body Composition

FPR has previously been used as an additive to supplement daily fish iron requirements [44,45]. Studies have shown that inorganic iron supplementation in the diet can increase the growth performance of some aquatic animals, such as bighead carp (*Aristichthys nobilis*), Indian major carp (*Labeo rohita*), and Chinese mitten crab (*Eriocheir sinensis*) [46,47,48]. However, our current result has shown that dietary FPR supplementation could not affect the growth performance of gibel carp, which was similar to the result on Atlantic salmon (*Salmo salar*) [44]. The differences could be due to the different biological efficacy of hemin-enriched organic and inorganic iron [49]. Moreover, the results of this experiment indicated that the addition of FPR had no effect on the whole-body protein and ash content compared to the control group. The group supplemented with 0.03% FPR exhibited the lowest whole-body lipid content and a significant increase in whole-body moisture. However, interestingly, the study on Indian major carp showed that dietary iron supplementation decreased the whole-body moisture and improved the whole-body lipid content [47]. In fish, there is a strong negative correlation between whole-body moisture and lipids. Increased moisture content found in this study may be attributed to the high levels of FPR inducing the expression of the HO-1 enzyme, which in turn decreases the whole-body lipid content and increases the whole-body moisture content [50]. However, the effect of FPR on fish body composition remains unclear, and further investigation is needed.

### 4.2. Effects of Dietary Supplementation with FPR on Plasma Parameters

Plasma parameters were usually used as important indices of fish health and physiological condition [51]. Plasma TP and ALB are associated with fish immune response [52,53], with increasing levels correlated with a stronger innate immune response [54]. The results of the present study demonstrated that 0.01% FPR supplementation significantly increased plasma TP and ALB levels, suggesting an enhanced immune response in gibel carp. Additionally, ALT and AST are sensitive indicators of liver function damage [55]. The results found that 0.03% and 0.04% FPR supplementation increased plasma ALT and AST levels, suggesting that high FPR levels may impair the liver health of gibel carp. These results are similar to studies that found that supplementation with high dietary iron increased the ALT and AST of largemouth bass (*Micropterus salmoides*) [49]. In this study, 0.01% FPR increased TC levels. This result coincides with another study that reported elevated TC levels in snow trout (*Schizothorax zarudnyi*) and rohu (*Labeo rohita*) following nanoparticulate iron supplementation [56,57]. However, this study found that the addition of 0.04% FPR significantly reduced TC levels. Prior research has demonstrated that the incorporation of FPR into the diet alters insulin sensitivity, thereby influencing fat and glucose metabolism in obese mice [58]. The same results were observed in this study, where the incorporation of 0.03% and 0.04% FPR led to a notable decline in TG and GLU levels. The specific reason is that high FPR can induce heme oxygenase enzymes expression and regulate the insulin sensitivity and lipid metabolism, and then decrease the accumulation of lipids and glucose [59].

### 4.3. Effects of Dietary Supplementation with FPR on Antioxidant Status

The primary mechanism driving oxidative stress is an imbalance between ROS and antioxidants [60]. The liver is an important organ that produces ROS and is susceptible to ROS exposure. However, the liver has robust antioxidant defense mechanisms that maintain ROS balance at physiological levels [61], as well as maintenance of other physiological processes, including cellular proliferation and immune response modulation [62]. A significant corpus of evidence demonstrates that the Nrf2 signaling pathway plays a key role in liver pathophysiology and is an important antioxidant defense system against oxidative stress injury by regulating gene expression [63,64]. Supplementation with high concentrations of FPR significantly suppressed the Nrf2 signaling pathway. Supplementation with 0.03% FPR significantly down-regulated the *nrf2* mRNA expression level, and the *keap1* mRNA expression level showed the same trend. This result may be attributed to the regulatory properties of the body in response to inflammation and reduced antioxidant enzyme activity [51,65]. Simultaneously, supplementing with 0.03% FPR significantly down-regulated expression levels of *cat* and *gpx* mRNA. These results indicate that high levels of FPR decreased the antioxidant functions of gibel carp, similar to results found in largemouth bass that showed that high-level supplementary iron suppressed the Nrf2 signaling pathway via oxidative stress [66]. Nevertheless, FPR supplementation did not result in alterations to *sod* expression levels. It is well-established that antioxidant enzyme activity is positively correlated with antioxidant gene expression [67]. As observed in this study, dietary supplementation of 0.03% and 0.04% FPR significantly decreased the activity of GPx. In addition, 0.03% and 0.04% dietary FPR significantly increased the MDA content, which is the product of lipid peroxidation during oxidative stress and is an important indicator of oxidative damage [68]. A potential mechanism underlying these results is the iron component in FPR. Excessive free iron stimulates the formation of ROS via the Fenton reaction to destroy the balance between ROS and antioxidants [69]. These results provide further evidence that high levels of FPR (0.03–0.04%) potentially reduce antioxidant enzyme activity and gene expression, enhance lipid peroxidation levels, and induce oxidative stress, undermining the antioxidant balance.

### 4.4. Effects of Dietary Supplementation with FPR on Oxygen Carrying Capacity

Hypoxia-inducible factor (HIF-1) is a transcription factor consisting of hypoxia-inducible factor-1α (HIF-1α) and arylhydrocarbon receptor nuclear translocator (HIF-1β) proteins [70]. The activation of HIF-1 induces the transcription of genes linked to apoptosis, erythropoiesis, and angiogenesis, which contribute to the maintenance of oxygen homeostasis in animals [71]. HIF-1α primarily determines the availability of HIF-1. In normoxic conditions, HIF-1α is maintained at low levels by controlled degradation, primarily through the VHL-dependent ubiquitin–proteasome pathway [72]. However, some studies have shown that HIF-1α can be induced by some growth factors, iron metabolism, and hormones under normal aerobic conditions [73]. In the current experiment, the expression level of *hif-1α* mRNA was not significantly altered by FPR supplementation. Nevertheless, supplementation of FPR at concentrations of 0.03% to 0.04% resulted in a notable reduction in the mRNA expression level of the downstream factor *epo*, which is a hematopoietic cytokine primarily known for its role in promoting the formation and survival of red blood cells [74]. Studies have reported similar results, demonstrating that FPR significantly decreased *epo* mRNA expression levels under normal aerobic conditions [75]. The decline in *epo* mRNA expression levels suggests that a higher proportion of FPR supplementation (0.03–0.04%) could limit the generation of red blood cells and reduce the oxygen transport capacity in gibel carp. In addition, increasing evidence indicates that oxidative-stress-induced increase in ROS promotes vascular endothelial growth factor (VEGF) synthesis, which is an important factor regulating angiogenesis and is synthesized in vast quantities in various inflamed tissues [76,77]. In this study, when supplemented with 0.04% FPR, the *vegf* mRNA expression level was significantly elevated, indicating that oxidative stress induced by the addition of high levels of FPR results in liver damage. This upregulation of *vegf* mRNA levels may be driven by iron-induced oxidative stress, a major component of FPR, which increases the formation of ROS, in turn stimulating the synthesis of VEGF. Jazwa used FPR to stimulate HaCaT keratinocytes and reported an increase in *vegf* mRNA expression [78]. However, the impact of FPR on the HIF-1α signaling pathway is intricate and necessitates further investigation.

### 4.5. Effects of Dietary Supplementation with FPR on Immunocompetence

Fish utilize the innate immune system as a defense mechanism against waterborne pathogens [79]. When cells are stimulated by external factors, the immune system is activated, leading to enhanced inflammatory factor levels [80]. The NF-κB signaling pathway is of great importance in the innate immune response, regulating a multitude of genes involved in the inflammatory response, including pro-inflammatory *il-1β*, *il-6*, and *tnf-α*, as well as anti-inflammatory *il-10* and *tgf-β* [81]. In the current study, 0.02% FPR supplementation significantly decreased the expression level of *nf-κb* mRNA compared to the control group. Additionally, the expression levels of downstream cytokines *il-1β* and *il-6* mRNA were significantly reduced by the addition of 0.01% and 0.02% FPR, respectively. The down-regulation of pro-inflammatory mRNA transcripts associated with the NF-κB pathway indicated that appropriate dietary supplementation of FPR between 0.01 and 0.02% may enhance the immune response of gibel carp. Similar results were found in a study examining largemouth bass [80], which demonstrated that supplementation with 60 mg/kg iron improved the immune capacity. In addition, the present study showed that supplementation with 0.02% FPR significantly up-regulated the expression levels of *il-10* mRNA, further highlighting that FPR may potentially improve the inflammatory function and immune response in gibel carp. However, the addition of higher concentrations (0.03–0.04%) was detrimental to immune function, as it inhibited the antioxidant capacity and increased the levels of ALT and AST, which in turn adversely affected liver health. As Chen [66] and He [80] reported, supplementing diets with excessive iron decreased the immunity of fish.

## 5. Conclusions

The results of this experiment demonstrated that the incorporation of 0.01–0.02% FPR into the feed increased the immune response of gibel carp. Lower concentrations of dietary FPR (0.01–0.02%) down-regulated the NF-κB signaling pathway, thereby improving the immune ability. However, supplementation with higher levels of FPR (0.03–0.04%) inhibited the Nrf2 and HIF-1α signaling pathways, leading to decreased antioxidant and oxygen-carrying capacity.

## Figures and Tables

**Table 1:** Formulation and proximate composition of diets in the experiment (% dry basis).

Ingredients (g/kg)	Level (%)	Ingredients (g/kg)	Level (%)
Fish meal ^1^	14.00	Calcium dihydrogen phosphate	2.00
Chicken meal ^1^	4.00	Vitamin premix ^2^	0.20
Soybean meal ^1^	22.00	Mineral premix ^2^	2.00
Cottonseed meal ^1^	5.00	Lysine ^3^	0.30
Rapeseed meal	22.00	Methionine ^3^	0.10
Wheat flour ^1^	14.15	L-Ascorbate-2-monophosphate	0.05
Rice bran	10.00	Choline chloride	0.20
Soybean oil	4.00		
Analyzed proximate composition (dry matter)			
Crude protein (%)	39.94 ± 0.29		
Crude lipid (%)	7.63 ± 0.39		
Crude ash (%)	10.02 ± 0.09		
Dry matter (%)	92.27 ± 0.12		
Energy (KJ)	13.75 ± 0.189		

Note: ^1^ All ingredients in this experiment were procured from Tongwei Feedstuffs Co. Ltd. (Wuxi, China). ^2^ All premixes in this experiment were sourced from HANOVEA Animal Health Products Co., Ltd. (Wuxi, China). ^3^ This experimental L-lysine and L-methionine were procured from Feeer Co., Ltd. (Shanghai, China).

**Table 2:** The R^2^ and PCR amplification efficiencies of the qPCR primer sequences and standard curves pertinent to this experiment.

Gene	Forward Primer (5′-3′)	Reverse Primer (5′-3′)	R^2^ (%)	PCR Efficiency	Accession Number/Reference
^1^ *β-actin*	GATGATGAAATTGCCGCACTG	ACCGACCATGACGCCCTGATGT	99.8	101.3	Yang [40]
^2^ *keap1*	CTCCGCTGAATGCTACAA	GGTCATAACACTCCACACT	99.7	104.8	XM_026245355.1
^3^ *nrf2*	TACCAAAGACAAGCAGAAGAAACG	GCCTCGTTGAGCTGGTGTTTGG	99.6	100.5	Sun et al. [41]
^4^ *sod*	TCGGAGACCTTGGTAATGT	CGCCTTCTCATGGATCAC	99.3	104.4	JQ776518.1
^5^ *cat*	TGAAGTTCTACACCGATGAG	CTGAGAGTGGACGAAGGA	99.1	101.9	XM_026238665.1
^6^ *gpx*	GAAGTGAACGGTGTGAACGC	GATCCCCCATCAAGGACACG	99.5	97.9	DQ983598.1
^7^ *nf-kb*	GCTCTGACTGCGGTCTTATAC	GCGCTTCATCGAGGATAGTT	99.3	102.5	Gu et al. [42]
^8^ *tgf-β*	GTTGGCGTAATAACCAGAAGG	AACAGAACAAGTTTGTACCGATAAG	99.6	99.0	Yang [40]
^9^ *il-10*	AGTGAGACTGAAGGAGCTCCG	TGGCAGAATGGTGTCCAAGTA	99.7	101.8	KHIEOKHAJONKHET [43]
^10^ *il-6*	CGGAGGGGCTTAACAGGATG	GCTGGCTCAGGAATGGGTAT	99.0	102.9	DQ861993.1
^11^ *tnf-α*	CATTCCTACGGATGGCATTTACTT	CCTCAGGAATGTCAGTCTTGCAT	99.7	98.1	Yang [40]
^12^ *il-1β*	GCGCTGCTCAACTTCATCTTG	GTGACACATTAAGCGGCTTCA C	99.4	101.3	Yang [40]
^13^ *hif-1α*	CTGCCGATCAGTCTGTCTCC	TTTGTGGAGTCTGGACCACG	99.8	105	DQ306727.
^14^ *epo*	CGAAGTGTCAGCATACCGGA	GCAGATGACGCACTTTTCCC	99.0	101.9	KC460317.1
^15^ *vegf*	ATCGAGCACACGTACATCCC	CCTTTGGCCTGCATTCACAC	99.0	102.8	NM_131408.3
^16^ *et1*	TAAAGCAGCGTCAGACAGGG	CTGCCAGCTTGTGTTTGCAT	99.5	99.6	NM_131519.1

Note: ^1^ *β-actin*, beta-actin; ^2^ *keap1*, Kelch-like ECH-associated protein 1; ^3^ *nrf2*, nuclear factor erythroid2-related factor 2; ^4^ *sod*, superoxide dismutase; ^5^ *cat*, catalase; ^6^ *gpx*, glutathione peroxidase; ^7^ *nf-kb*, nuclear factor kappa-B; ^8^ *tgf-β*, transforming growth factor-β; ^9^ *il-10*, interleukin-10; ^10^ *il-6*, interleukin-6; ^11^ *tnf-α*, tumor necrosis factor-α; ^12^ *il-1β*, interleukin-1β; ^13^ *hif-1α*, hypoxia-inducible factor-1α; ^14^ *epo*, erythropoietin; ^15^ *vegf*, vascular endothelial growth factor; ^16^ *et1*, endothelin.

**Table 3:** Effect of dietary FPR levels on the growth performance of gibel carp fed the experimental diet for 8 weeks.

Dietary FPR Level (%)	^1^ IW (g)	^2^ FW (g)	^3^ WGR (%)	^4^ SGR (%/d)	^5^ FCR	^6^ SR (%)
0	36.4 ± 0.07	87.0 ± 2.18	140 ± 5.76	0.94 ± 0.03	1.21 ± 0.05	100 ± 0.00
0.01	36.3 ± 0.08	89.3 ± 4.45	146 ± 12.67	0.96 ± 0.05	1.17 ± 0.09	100 ± 0.00
0.02	36.4 ± 0.12	89.7 ± 0.72	147± 2.21	0.97 ± 0.01	1.15 ± 0.01	100 ± 0.00
0.03	36.4 ± 0.16	90.7 ± 1.04	149 ± 1.94	0.98 ± 0.01	1.13 ± 0.02	100 ± 0.00
0.04	36.4 ± 0.11	84.2 ± 2.76	131 ± 7.04	0.90 ± 0.03	1.29 ± 0.07	100 ± 0.00

Note: The values are shown as mean ± SE. The subsequent parameters were derived through the application of the aforementioned methodology to the data obtained throughout the course of the trial. ^1^ Initial body weight (IW). ^2^ Final average weight (FW) (g). ^3^ Weight gain rate (WGR) (%) = 100 × (FW (g) − IW (g))/IW (g). ^4^ Specific growth rate (SGR) (%/day) = 100 × ((ln (FW (g) − ln (IW (g)))/days). ^5^ Feed conversion ratio (FCR) = dry feed fed (g)/(FW (g) − IW (g)). ^6^ Survival rate (SR) (%) = 100 × (final number of fish / initial number of fish).

**Table 4:** Effect of dietary FPR levels on the whole body of gibel carp fed the experimental diet for 8 weeks.

Dietary FPR Level (%)	Moisture (%)	Protein (%)	Lipid (%)	Ash (%)
0	74.2 ± 1.23 ^a^	16.6 ± 0.13	2.2 ± 0.27 ^ab^	4.8 ± 0.21
0.01	75.3 ± 0.50 ^ab^	15.9 ± 0.31	2.5 ± 0.63 ^ab^	4.8 ± 0.08
0.02	75.1 ± 0.53 ^ab^	15.2 ± 0.89	3.1 ± 0.48 ^b^	4.8 ± 0.08
0.03	77.2 ± 0.42 ^b^	15.4 ± 0.21	1.3 ± 0.13 ^a^	4.6 ± 0.16
0.04	74.3 ± 0.66 ^a^	16.3 ± 0.35	3.0 ± 0.85 ^ab^	4.8 ± 0.07

Note: The values are shown as mean ± SE. The appearance of superscripts in the same column with different letters denotes statistically significant disparities (*p* < 0.05), whereas the absence of superscripts signifies that the difference is not significant (*p* > 0.05).

**Table 5:** Plasma biochemical parameters.

Parameters	Supplement Levels (%)				
	0	0.01	0.02	0.03	0.04
^1^ ALB(mmol/L)	7.7 ± 0.43 ^a^	8.5 ± 0.13 ^b^	8.23 ± 0.21 ^ab^	7.6 ± 0.24 ^a^	8.3 ± 0.21 ^ab^
^2^ ALT(U/L)	0.78 ± 0.10 ^a^	0.82 ± 0.20 ^ab^	0.90 ± 0.13 ^ab^	1.27 ± 0.16 ^b^	1.14 ± 0.10 ^ab^
^3^ AST(U/L)	135 ± 6.21 ^a^	137 ± 6.54 ^a^	137 ± 7.99 ^a^	143 ± 6.48 ^a^	169 ± 7.63 ^b^
^4^ TC(mmol/L)	5.7 ± 0.12 ^b^	6.2 ± 0.16 ^c^	5.7 ± 0.06 ^b^	5.5 ± 0.11 ^b^	5.2 ± 0.12 ^a^
^5^ TG (mmol/L)	1.35 ± 0.06 ^a^	1.26 ± 0.03 ^ab^	1.23 ± 0.03 ^ab^	1.21 ± 0.04 ^b^	1.16 ± 0.05 ^b^
^6^ GLU(mmol/L)	8.95 ± 0.26 ^ab^	9.19 ± 0.44 ^a^	8.29 ± 0.13 ^b^	6.81± 0.29 ^c^	6.98 ± 0.16 ^c^
^7^ TP(g/ L)	26.7 ± 0.76 ^a^	28.4 ± 0.53 ^b^	26.1 ± 0.38 ^a^	25.8 ± 0.44 ^a^	26.2 ± 0.64 ^a^

Note: Mean values are shown as mean ± SE. ^1^ ALB, albumin. ^2^ ALT, alanine aminotransferase. ^3^ AST, aspartate aminotransferase. ^4^ TC, total cholesterol. ^5^ TG, total triglyceride. ^6^ GLU, glucose. ^7^ TP, total protein. The appearance of superscripts in the same row with different letters denotes statistically significant disparities (*p* < 0.05), whereas the absence of superscripts signifies that the difference is not significant (*p* > 0.05).

## Data Availability

Data are contained within the article.
